# Health outcomes in a national sample of American Indian and Alaska Native adults: Differences between multiple-race and single-race subgroups

**DOI:** 10.1371/journal.pone.0242934

**Published:** 2020-12-03

**Authors:** Ursula Running Bear, Nancy L. Asdigian, Janette Beals, Spero M. Manson, Carol E. Kaufman

**Affiliations:** 1 Department of Population Health, School of Medicine and Health Sciences, University of North Dakota, Grand Forks, ND, United States of America; 2 Department of Community and Behavioral Health, Centers for American Indian and Alaska Native Health, Colorado School of Public Health, University of Colorado Anschutz, Medical Campus, Aurora, CO, United States of America; Indiana University Purdue University at Indianapolis, UNITED STATES

## Abstract

**Objectives:**

To determine differences among multi-race (MR) American Indian and Alaska Natives (AIAN), single race (SR) AIANs, and SR-Whites on multiple health outcomes. We tested the following hypotheses: MR-AIANs will have worse health outcomes than SR-AIANs; SR-AIANs will have worse health outcomes than SR-Whites; MR-AIANs will have worse health outcomes than SR-Whites.

**Methods:**

Behavioral Risk Factor Surveillance System data were used to examine general health, risk behaviors, access to health care, and diagnosed chronic health conditions. Those identifying as SR-White, SR-AIAN, and MR-AIAN were included in multinomial logistic regression models.

**Results:**

Compared to SR-AIANs, MR-AIANs had more activity limitations, a greater likelihood of experiencing cost as a barrier to health care and were more likely to be at increased risk and diagnosed with more chronic health conditions. Both SR and MR-AIANs have worse health than SR-Whites; MR-AIANs appear to be at increased risk for poor health.

**Conclusions:**

The current study examined access to health care and nine chronic health conditions, neither of which have been considered in prior work. MR AIANs are at increased risk compared to SR groups. These observations beg for further inquire into the mechanisms underlying these differences including stress related to identify, access to care, and discrimination. Findings support the continued need to address health disparities among AIANs regardless of SR or MR identification.

## Introduction

American Indian/Alaska Native (AIAN) people are disproportionately burdened by poor health and comprise one of the largest multi-race (MR) groups in the US [1]. Identifying health risks and outcomes related to MR-AIAN status will aid our understanding of the ensuing health disparities. A small body of research shows that people who identify as MR report poorer mental and physical health outcomes compared to their single race (SR) counterparts [2, 3]. The specific mechanisms underlying such differences are not well understood, but several possibilities have emerged that converge on stress as a common factor. First, MR people may experience uncertainty or confusion about their identity and thus have difficulty describing themselves in a manner consistent with their self-image [4]. Another possibility is MR people may experience a discrepancy between their self-ascribed race and the race attributed to them by others [5]. Lastly, introduced by Park in 1928 is the concept of the “marginal man,” where the MR person lives in two or more distinct cultures but is an outsider to all [6]. The shared premise among these perspectives is that MR people are more likely than SR people to experience stress related to their racial identity, which may increase the risk for a number of health conditions [2, 7].

In addition to stress related to identity, other factors may contribute to poor MR-AIAN health outcomes such as access to care and discrimination. The Indian Health Service (IHS) provides health care to federally recognized AIANs in the US [8]. Although, approximately 70% of AIANs reside in urban areas only 41 urban IHS funded facilities exist nationwide [8]. The result is many urban areas lack IHS funded facilities leaving eligible AIANs without healthcare. Prior research demonstrated that AIANs with access to IHS heath care were more likely to receive health services than AIANs without IHS access [9]. Racial discrimination impacts both the ability to receive health care and health outcomes. More than one in five AIANs reported discrimination during clinical encounters and, due to anticipated discrimination, 15% avoid health care for either themselves or family [10]. Similarly, AIANs visiting an urban pediatric emergency department were less likely to have received radiology testing compared to White patients [11]. Discrimination is also associated with health outcomes like cardiovascular disease for many racial groups [12] and high blood pressure in American Indians (AI) [13]. The premise behind these studies is that discrimination is associated with increases in stress hormones like cortisol, heart rate variability, and higher allostatic load [13].

Growing evidence supports the association of MR identity with poor health outcomes. One study that aggregated MR people together regardless of racial combination observed, MR people were more likely to smoke and drink alcohol compared to SR groups (White, African, Asian Americans) [14]. Another study of chronic physical health symptoms among MR people, again regardless of racial combination, revealed they reported more symptoms than SR-Asians and SR-Whites [7]. Similar patterns are evident among American Indian and Alaska Natives (AIANs). MR AI-White youth were more likely to report poor health, bodily aches and pains, higher rates of depression, and smoke and drink regularly compared to SR-White and SR-AI youth [2]. Our previous work indicated MR-AIANs exhibit a higher prevalence of lifetime depression, more days of poor mental health, and more frequent mental distress compared to SR-AIANs and SR-Whites [3]. Others, focused solely on a unitary measure of health, observed that MR AI-White and MR AI-Black adults were both more likely to report poor self-rated health compared to SR-Whites [15]. They also found that SR-AIs reported poorer self-rated health compared to SR-Whites but better self-rated health than MR AI-Whites and MR AI-Blacks [15]. Lastly, one study found MR AI-s were at increased risk for respiratory illnesses and had higher rates of asthma, hay fever, sinusitis, and chronic obstructive pulmonary disease than SR-AIs and SR-Whites [16]. These findings support a thin layer of evidence suggesting MR status may be an indicator of compromised health, yet access to health care, risk for chronic conditions, as well as specific general measures like activity limitations, and behavioral risk factors such as obesity and exercise have not been considered.

We examined differences between MR-AIANs, SR-AIANs, and SR-Whites on health outcomes across four categories: general health, behavioral risk factors, health care access, and chronic health conditions. Drawing upon the existing literature summarized above, we posited three hypotheses: 1) MR-AIANs will have worse health outcomes compared to SR-AIANs; 2) SR-AIANs will have worse health outcomes compared to SR-Whites; and 3) MR-AIANs will have worse health compared to SR-Whites.

## Methods

### Data source

The BRFSS survey is recognized as a leading source of health data. It is the largest continuously conducted survey in the world that focuses on non-institutionalized adults aged 18 or older and inquiries about health practices, health risks, and chronic health conditions [17]. Its national telephone interview (landline and cellular) is conducted annually in collaboration with each state and the Centers for Disease Control and Prevention [18]. Designed as a complex survey sample, a method of weighting the sample known as raking, accounts for sample selection and non-response which increases the representation of population subgroups otherwise underrepresented in the sample [18]. Although a multiple race question is now standard, after 2012, detailed data on the multi-race responses are no longer available in the public use data. Therefore, we used data from the 2012 dataset to conduct the analyses reported here. We limited our BRFSS analyses to respondents within the 50 states and the District of Columbia (DC) who self-identified as either SR-White, SR-AIAN, or AIAN in combination with one or more other races (MR-AIAN).

### Availability of data

BRFSS data were used for these analyses. The data analyzed for this study are publicly and freely available at the BRFSS website located here, https://www.cdc.gov/brfss/annual_data/annual_2012.html. Our team used the publicly available data accessible at this location. We did not request or use data that required special approval or access.

### Measures

We drew upon responses from the BRFSS 2012 core questions asked of all respondents. Our analyses included self-reported demographics, general health variables, risk behaviors, access to health care, and diagnosed chronic health conditions.

The question, “Which one or more of the following would you say is your race? (Check all that apply) White, Black or African American, Asian, Native Hawaiian or Other Pacific Islander, American Indian or Alaska Native, and Other (specify)”, was used to construct the MR variable. We developed three categories, SR-AIAN, MR-AIAN, and SR-White. Respondents selecting White only or AIAN only from among these options comprised the SR groups. Those indicating AIAN in combination with any other race or combination of races comprised the MR-AIAN category.

General health assessments included poor health, frequent distress, and activity limitations. We constructed these variables similarly to other studies [19–27]. Poor health status derived from the question that asked, “Would you say that in general your health is excellent, very good, good, fair, or poor?” Those selecting fair or poor were designated as having poor health [19, 23]. Frequent distress was constructed from the question “During the past 30 days, for about how many days did poor physical or mental health keep you from doing your usual activities, such as self-care, work, or recreation?” Following general convention [20, 21, 23], respondents who indicated ≥14 days were coded as being in frequent distress. Activity limitations were defined by a yes answer to the question “Are you limited in any way in any activities because of physical, mental, or emotional problems?” [20, 21].

We considered five behavioral risk factors: obesity, lack of exercise, current smoker, binge drinking, and heavy drinking. Respondents with a body mass index (BMI) ≥30 were designated as obese [22]. Lack of exercise was defined as not participating in any physical activity or exercise in the past 30 days [20, 25]. Individuals answering either “every day” or “at least some days” to the question “Do you now smoke cigarettes?” were designated as current smokers [24, 25] Binge drinkers were defined as those who, on at least one occasion in the past 30 days, drank five drinks during one occasion for men and four drinks for women [22, 24].Heavy drinking for men was defined as drinking two drinks per day and one drink per day for women in the past 30 days [25, 27].

Two access to health care variables were also included. Respondents answering “no” to the question, “Do you have any kind of health care coverage, including health insurance, prepaid plans such as HMOs, government plans such as Medicare or Indian Health Service?” were identified as lacking health care coverage [22, 24]. Those answering yes to the question, “Was there a time in the past 12 months when you needed to see a doctor but could not because of cost?” were coded as wanting to see a doctor but did not because of cost [20, 26].

Nine chronic health conditions were included in our analyses. Yes, to the question “Has a doctor, nurse, or other health professional EVER told you that you had any of the following?” defined the presence of: 1) cardiovascular disease (combined heart attack, myocardial infarction, coronary heart disease, and stroke) [19, 21]; 2) skin cancer; 3) other cancers; 4) lung disease (combined chronic obstructive pulmonary disease, emphysema, chronic bronchitis, asthma), 5) arthritis, 6) depressive disorder (including depression, major depression dysthymia, minor depression), 7) kidney disease, and 8) diabetes. We created a ninth variable; comorbid disorders indicated whether two or more chronic conditions were present.

We controlled for demographic variables which included Hispanic ethnicity, sex (male, female), six age categories (18–29, 30–39, 40–49, 50–59, 60–69, and 70 years or older), marital status (married, cohabiting, separated/widowed/divorced, never married), children less than 18 years of age in the home (0, 1–2, 3–4, 5 or more), education (less than high school, high school, some college, college graduate), in the labor force, income (less than $15K per year, $15-$24.9K, $25-$34.9K, $35-$49.9K, $50K or more, missing), and region of residence (East, Northern Plains, Southwest, Pacific, Southern Plains, Alaska). To examine AIAN regional variation we constructed a region of residence variable similar to other studies using BRFSS data [28]. We defined geographical regions as follows: **East** (Alabama, Arkansas, Connecticut, Delaware, Florida, Georgia, Kentucky, Louisiana, Maine, Maryland, Massachusetts, Mississippi, Missouri, New Hampshire, New Jersey, New York, North Carolina, Ohio, Pennsylvania, Rhode Island, South Carolina, Tennessee, Vermont, Virginia, West Virginia, and the District of Columbia), **Northern Plains** (Illinois, Indiana, Iowa, Michigan, Minnesota, Montana, Nebraska, North Dakota, South Dakota, Wisconsin, and Wyoming), **Southwest** (Arizona, Colorado, Nevada, New Mexico, and Utah), **Pacific** (California, Idaho, Oregon, Washington, and Hawaii), **Southern Plains** (Kansas, Oklahoma, and Texas), and the state of Alaska comprised the **Alaska** region. Metropolitan statistical area (MSA) status was operationalized as either not in an MSA or county with an MSA. MSA was defined as in the center city of an MSA, outside the center city of an MSA but inside the county containing the center city, inside a suburban county of the MSA, or in an MSA that has no center city.

### Research ethics

The Colorado Institutional Review Board determined these analyses were not human subjects research. The University of North Dakota did not require a review since Dr. Running Bear’s analyses were completed at the University of Colorado Anschutz Medical Campus, prior to employment at the University of North Dakota.

## Analytic approach

All analyses were weighted and Stata was used to produce estimates [29]. Descriptive statistics represent the percentage of individuals in each race group endorsing each sociodemographic variable. Using methods refined by Long and Freese specific to Stata [30], multinomial logistic regression methods (MNLR) were used to assess whether the three race groups differed on the health variables examined. Stata MNLR procedures allowed for pairwise comparisons among the race groups reported (SR-AIAN vs MR-AIAN, SR-AIAN vs SR-White, MR-AIAN vs SR-White).

The income and MSA variables displayed large proportions of missing data, 13% and 41% percent, respectively. The MSA variable was collected for respondents that had a land line. Individuals completing the survey by cellphone are missing the MSA variable which accounts for the large amount of missing data. We chose to create a missing category for the MSA variable because a larger proportion of AIANs both SR and MR appear to have participated by cell phone. The indicator variable for missing MSA data was used as a covariate in all analyses. Following the approach of others we created a missing data category for income that was included in all analyses [20]. Once accounting for the missing data in the MSA and income variables the entire dataset had approximately 2% missing data.

## Results

Of the 475,687 respondents in the BRFSS 2012 data, we identified 393,681 (82.7%) who were either SR-AIAN, MR-AIAN, or SR-White and lived within the 50 states or DC. Of these, 7,976 (2.0%) identified as SR-AIAN, 5,512 (1.4%) as MR-AIAN, and 380,193 (96.6%) as SR-White. Approximately, two-thirds (64%) of the MR-AIAN group chose White as the race that best represented them; 818 (15%) selected AIAN; the remaining 1,236 (21%) selected one of the other races (Black or African American, Asian, Native Hawaiian or Pacific Islander, Other).

### Demographic variables

Table 1 reports percentages for each demographic variable and race group, as well as unadjusted MNLRs for each variable and pairwise race comparison. SR-AIANs were younger than both MR-AIANs and SR-Whites. SR-AIANs had more children in the home compared to MR-AIANs and SR-Whites. Fewer SR-AIANs attended college than either MR-AIANs or SR-Whites. Both MR-AIANs and SR-AIANs were less likely to be in the labor force compared to SR-Whites. More SR-AIANs reported incomes at or below $24.9K than either MR-AIANs and SR-Whites. A larger proportion of SR-AIANs reside in the Pacific region whereas a larger proportion of MR-AIANs and SR-Whites reside in the East. More SR-AIANs had missing MSA data than did MR-AIANs and SR-Whites. Fewer SR-AIANs, compared to MR-AIANs and SR-Whites lived within an MSA.

**Table 1:** Demographics by race group.

	DESCRIPTIVE STATISTICS						MULTINOMIAL LOGISTIC REGRESSION							
	SR AIAN		MR AIAN		SR White		OMNIBUS^a^		Odds Ratio^b^					
N	7,976		5,512		380,193		F	p						
	%	CI	%	CI	%	CI			SR AIAN v MR AIAN		SR AIAN v SR White		MR AIAN v SR White	
**Hispanic Ethnicity**	41%	[38%, 44%]	16%	[14%, 19%]	12%	[12%, 13%]	311.25	0.0001	3.65	****	4.92	****	1.35	***
**Male**	55%	[52%, 58%]	51%	[48%, 54%]	48%	[48%, 49%]	13.63	0.0001	1.16	^ ^	1.31	****	1.13	*
**Age groups**							15.82	0.0001		^ ^		^ ^		^ ^
18 to 29	27%	[25%, 30%]	22%	[20%, 25%]	19%	[19%, 20%]			2.11	****	2.98	****	1.42	**
30 to 39	20%	[17%, 22%]	17%	[15%, 19%]	15%	[15%, 16%]			2.00	****	2.70	****	1.35	*
40 to 49	17%	[15%, 19%]	15%	[13%, 18%]	17%	[17%, 17%]			1.86	****	2.08	****	1.12	^ ^
50 to 59	19%	[17%, 21%]	19%	[17%, 21%]	19%	[19%, 19%]			1.69	****	2.07	****	1.22	^ ^
60 to 69	11%	[10%, 13%]	15%	[13%, 17%]	15%	[15%, 15%]			1.26		1.58	****	1.26	*
70 and older	7%	[6%, 8%]	11%	[10%, 14%]	14%	[14%, 14%]			1.0						
**Marital status**							29.58	0.0001				^ ^		^ ^
Married/cohabiting	49%	[46%, 52%]	47%	[44%, 50%]	59%	[59%, 59%]			1.0						
Separated/divorced/widowed	23%	[21%, 25%]	27%	[24%, 29%]	20%	[20%, 20%]			0.82	*	1.40	****	1.70	****
Never married	28%	[26%, 31%]	27%	[24%, 30%]	21%	[21%, 22%]			1.03		1.60	****	1.56	****
**Children in the home**							24.57	0.0001		^ ^		^ ^		^ ^
None	51%	[48%, 54%]	65%	[62%, 68%]	65%	[65%, 66%]			1.0					
1 or2	34%	[31%, 37%]	26%	[24%, 29%]	27%	[27%, 27%]			1.66	****	1.63	****	0.98	^ ^
3 or4	13%	[11%, 15%]	7%	[6%, 9%]	7%	[7%, 7%]			2.32	****	2.29	****	0.99	^ ^
5 or more	3%	[2%, 4%]	1%	[.8%, 2.2%]	1%	[.8%, .9%]			2.49	**	4.08	****	1.64	^ ^
**Education**							121.35	0.0001				^ ^		^ ^
Less than high school	32%	[29%, 35%]	18%	[15%, 21%]	13%	[13%, 13%]			1.0					
High school	33%	[31%, 36%]	28%	[26%, 31%]	29%	[29%, 30%]			0.66	**	0.46	****	0.70	***
Some college	26%	[24%, 28%]	36%	[33%, 39%]	31%	[31%, 31%]			0.40	****	0.34	****	0.84	^ ^
College graduate	9%	[8%, 10%]	18%	[16%, 20%]	27%	[27%, 27%]			0.29	****	0.14	****	0.49	****
**In the labor force**	49%	[46%, 52%]	47%	[44%, 50%]	56%	[56%, 56%]	28.47	0.0001	1.10	^ ^	0.76	****	0.69	****
**Income**							61.10	0.0001				^ ^		^ ^
Less than $15K	24%	[22%, 26%]	18%	[16%, 21%]	9%	[09%, 10%]			1.0					
$15 to $24.9K	21%	[19%, 23%]	18%	[16%, 21%]	14%	[14%, 15%]			0.86		0.58	****	0.68	****
$25 to $34.5K	12%	[10%, 14%]	11%	[9%, 13%]	9%	[9%, 10%]			0.85		0.50	****	0.59	****
$35 to $49.9K	10%	[9%, 12%]	12%	[10%, 14%]	13%	[13%, 13%]			0.67	**	0.32	****	0.49	****
$50K or more	18%	[16%, 20%]	28%	[25%, 30%]	41%	[41%, 42%]			0.50	****	0.18	****	0.35	****
Missing	15%	[13%, 18%]	13%	[11%, 16%]	13%	[13%, 13%]			0.85		0.46	****	0.54	****
**Geographical region of residence**							168.64	0.0001		^ ^		^ ^		^ ^
East	29%	[27%, 32%]	40%	[37%, 43%]	51%	[51%, 51%]			1.0					
Northern Plains	12%	[11%, 14%]	14%	[13%, 17%]	17%	[17%, 17%]			1.16	^ ^	1.24	**	1.07	^ ^
Southwest	12%	[11%, 13%]	5%	[4%, 6%]	6%	[6%, 6%]			3.22	****	3.25	****	1.01	^ ^
Pacific	31%	[28%, 34%]	25%	[22%, 27%]	16%	[16%, 16%]			1.72	****	2.28	***	1.97	****
Southern Plains	14%	[12%, 16%]	15%	[13%, 18%]	10%	[10%, 10%]			1.24	^ ^	2.44	****	1.97	****
Alaska	2%	[1.6%, 2.0%]	1%	[.05%, .08%]	.2%	[.2%, .2%]			3.74	****	14.63	****	3.93	****
**Metropolitan Status Code**							25.76	0.0001		^ ^		^ ^		^ ^
Not in Metropolitan Statistical Area	14%	[13%, 15%]	11%	[10%, 13%]	14%	[14%, 14%]			1.0					
Within Metropolitan Statistical Area	42%	[39%, 45%]	48%	[45%, 51%]	53%	[53%, 53%]			0.71	****	0.79	****	1.10	^ ^
Missing	44%	[41%, 46%]	41%	[38%, 44%]	33%	[33%, 33%]			0.85	^ ^	1.31	****	1.54	****

a This is the overall F for the bivariate MNLR which tells us if the 3 groups differ on the demographic variable.

b These are bivariate OR's where each group is compared with the other.

* p < .05

** p < .01

*** p < .001

**** p < .0001

Table 2 reports descriptive statistics for each race group and health outcome, the unadjusted MNLR results for each pairwise comparison and each health outcome, and the adjusted MNLR for each health variable and racial pairwise comparison in the following categories: general health, behavioral risk factors, access to care, and chronic conditions. We limit the detailed reporting of our findings to the statistically significant MNLR adjusted results. Compared to MR-AIANs, SR-AIANs were 27% less likely to report activity limitations and 37% less likely to report that they wanted to see a doctor but couldn’t because of cost. SR-AIANs were 47% less likely to report skin cancer, 22% less likely to report lung diseases, 30% less like to report depressive disorders, and 23% less likely to report two or more chronic conditions compared to MR AIANs. No other significant differences were found between these two groups.

**Table 2:** Health indicators by race group.

	DESCRIPTIVES						MULTINOMIAL LOGISTIC REGRESSION														
	SR AIAN		MR AIAN		SR White		OMNIBUS		ORs												
	7,976		5,512		380,193		F^a^	p	Unadjusted						Adjusted for Sociodemographics^b^					
	%	CI	%	CI	%	CI			SR AIAN v MR AIAN		SR AIAN v SR White		MR AIAN v SR White		SR AIAN v MR AIAN		SR AIAN v SR White		MR AIAN v SR White	
**GENERAL HEALTH**																					
Poor health	29%	[27%, 32%]	27%	[25%, 31%]	17%	[17%, 17%]	105.81	0.0001	1.11	^ ^	2.08	****	1.88	****	0.92	^ ^	1.38	****	1.50	****
Frequent distress	14%	[12%, 15%]	17%	[15%, 20%]	8%	[8%, 8%]	81.83	0.0001	0.75	**	1.81	****	2.42	****	0.83	^ ^	1.41	****	1.70	****
Activity limitations	27%	[25%, 29%]	36%	[33%, 39%]	21%	[21%, 21%]	74.06	0.0001	0.65	****	1.38	****	2.12	****	0.73	**	1.36	****	1.85	****
**BEHAVIORAL RISK FACTORS**																					
Obese	34%	[32%, 37%]	34%	[31%, 37%]	27%	[27%, 27%]	26.83	0.0001	1.04	^ ^	1.42	****	1.37	****	0.99	^ ^	1.27	****	1.31	****
Lack of exercise	29%	[27%, 32%]	25%	[22%, 28%]	22%	[22%, 22%]	20.72	0.0001	1.26	*	1,48	****	1.17	*	1.12	^ ^	1.15	*	1.03	^ ^
Current smoker	29%	[26%, 31%]	29%	[27%, 33%]	19%	[19%, 19%]	71.56	0.0001	0.96	^ ^	1.73	****	1.80	****	0.96	^ ^	1.43	****	1.49	****
Binge drinking	19%	[16%, 22%]	18%	[15%, 21%]	18%	[17%, 18%]	0.49	0.6126	0.88	^ ^	0.64	****	0.72	****	1.08	^ ^	1.05	^ ^	0.97	^ ^
Heavy drinking	7%	[5%, 8%]	6%	[5%, 8%]	6%	[6%, 7%]	0.03	0.974	1.08	^ ^	1.09	^ ^	1.01	^ ^	1.22	^ ^	1.18	^ ^	0.97	*
**HEALTH CARE ACCESS**																					
Lack of health care coverage	28%	[26%, 31%]	22%	[19%, 25%]	16%	[16%, 16%]	58.73	0.0001	1.43	***	2.08	****	1.45	****	0.82	^ ^	0.80	**	0.98	^ ^
Wanted to see Dr. but didn't because of cost	24%	[22%, 26%]	27%	[24%, 30%]	15%	[14%, 15%]	91.53	0.0001	0.87	^ ^	1.84	****	2.12	****	0.62	****	1.00	^ ^	1.61	****
**CHRONIC CONDITIONS**																					
CVD (MI, CHD, stroke)	11%	[10%, 13%]	15%	[13%, 17%]	9%	[9%, 9%]	25.51	0.0001	0.74	**	1.33	****	1.80	****	0.87	^ ^	1.54	****	1.78	****
Skin cancer	2%	[2%,3%]	6%	[4%, 7%]	7%	[7%, 7%]	24.99	0.0001	0.38	****	0.29	****	0.76	^ ^	0.53	****	0.45	****	0.85	^ ^
Other cancers	5%	[5%, 6%]	8%	[7%, 10%]	7%	[7%, 7%]	6.24	0.0010	0.64	***	0.75	**	1.18	^ ^	0.87	^ ^	1.09	^ ^	1.26	*
Lung (COPD, emphysema, chronic bronchitis, asthma)	23%	[20%, 25%]	30%	[27%, 33%]	17%	[16%, 17%]	71.64	0.0001	0.69	****	1.46	****	2.11	****	0.78	*	1.48	****	1.90	****
Arthritis	26%	[24%, 28%]	38%	[35%, 41%]	27%	[27%, 28%]	27.71	0.0001	0.59	****	0.94	^ ^	1.60	****	0.74	**	1.30	****	1.75	****
Depressive disorder	20%	[18%, 22%]	28%	[26%, 31%]	18%	[18%, 18%]	36.55	0.0001	0.62	****	1.13	*	1.82	****	0.70	****	1.06	^ ^	1.57	****
Kidney disease	3%	[3%, 5%]	3%	[3%, 4%]	3%	[3%, 3%]	3.39	0.0336	1.09	^ ^	1.34	*	1.24	^ ^	1.19	^ ^	1.28	^ ^	1.08	^ ^
Diabetes	14%	[12%, 16%]	15%	[12%, 17%]	10%	[9%, 10%]	30.21	0.0001	0.96	^ ^	1.54	****	1.61	****	1.01	^ ^	1.50	****	1.49	****
Two or more chronic conditions	29%	[26%, 31%]	39%	[36%, 42%]	26%	[25%, 26%]	47.45	0.0001	0.64	****	1.16	**	1.83	****	0.77	**	1.42	****	1.84	****

a This is the overall F for the bivariate MNLR which tells us if the 3 groups differ on the health variable.

b These are bivariate OR's where each group is compared with the other.

* p < .05

** p < .01

*** p < .001

**** p < .0001

SR-AIANs were 38% more likely to report poor health, 41% more likely to experience frequent distress, 36% more likely to have activity limitations, 27% more likely to be obese, 15% more likely to lack physical activity, and 43% more likely to currently smoke than SR-Whites. SR-AIANs were also 54% more likely to report CVD, lung disease (48%), arthritis (30%), diabetes (50%), and have two or more chronic health conditions (42%) but 55% less likely to report skin cancer compared to SR-Whites.

Compared to SR-Whites, MR-AIANs were 50% more likely to report poor health, 70% more likely to report frequent distress, 85% more likely to have activity limitations, 31% more likely to be obese, 49% more likely to be a current smoker, and 61% less likely to want to see a doctor but couldn’t because of cost. MR-AIANs were also more likely to report CVD (78%), other types of cancer (26%), lung diseases (90%) arthritis (75%), depressive disorders (57%), diabetes (49%), and 2 or more chronic conditions (84%) compared to SR-Whites.

## Discussion

These analyses investigated differences in health outcomes between SR-AIANs and MR-AIANs as well as differences between these two groups and SR-Whites. Our first hypothesis, that SR-AIANs have better outcomes than MR-AIANs, was supported for seven of the 19 health conditions examined, five of these were chronic health conditions. No statistically significant differences were found between SR-AIANs and MR-AIANs in behavioral risk factors. Fewer differences in health outcomes were evident among SR and MR-AIANs, than for the other race comparisons.

Our second hypothesis was SR-AIANs will have worse health outcomes compared to SR-Whites. SR-AIANs had worse health outcomes for all general health variables, three of the five behavioral risk factors, and over half of the chronic conditions. However, fewer SR-AIANs reported skin cancer and lacked health care coverage compared to SR-Whites.

The last hypothesis postulated that MR-AIANs would report worse health outcomes than SR-Whites. Thirteen of the 17 indicators differed significantly between MR-AIANs and SR-Whites. Ten health outcomes were statistically significant when we compared both SR-AIANs and MR-AIANs to SR-Whites. They were poor health, frequent distress, activity limitations, obesity, current smoker, CVD, lung disorders, arthritis, depressive disorders, diabetes, and two or more chronic conditions.

Four differences emerged when comparing SR-AIANs and MR-AIANs to SR-Whites. SR-AIANs were more likely to lack exercise, though this was not statistically significant for MR-AIANs. This difference may be due to the lack of available and accessible physical activities on reservations [31]. Wanting to see a doctor but did not because of cost was statistically significant between MR-AIANs and SR-Whites, but not SR-AIANs. We suspect this difference may be due to SR-AIANs use of Indian Health Services for health care. Turning to the pairwise comparisons of SR-AIANs and MR-AIANs to SR-Whites, MR-AIANs fared worse with respect to the number of statistically significant health outcomes. Additionally, SR-AIANs were less likely to report skin cancer than SR-Whites while MR-AIANs were more likely to report other cancers compared to SR-Whites.

Our findings are consistent with previous studies that found MR-AIANs are at increased risk for poor health, frequent distress, smoking, and respiratory illnesses compared to SR-Whites. Clearly, failing to measure MR status in studies of AIAN health likely limits the insight to be gained into the differential risks, outcomes, and subsequent disparities [32]. Additionally, grouping MR-AIANs into a general MR category may obscure important variation across MR people. These observations beg for further inquiry into the specific mechanisms underlying such differences in health between SR and MR people.

While there is good reason to suspect stress related to MR identity may contribute to poor health, the biological stress response has yet to be carefully considered. Similar to studies that show discrimination is associated with conditions like high blood pressure or CVD, stress related to MR identify may also impact health. Comparisons of the biological stress response among MR-AIANs and SR-AIANs may help to clarify the contribution of stress related to racial identity.

Although no difference was observed between MR-AIANs and SR-Whites with respect to lack of health care coverage, MR-AIANs were more likely to have difficulty paying to see a doctor. SR-AIANs had lower incomes, were less likely to report higher education levels and were also less likely to have difficulty accessing a doctor than MR-AIANs. SR-AIANs also had slightly better health than MR-AIANs with respect to several outcomes. We can only theorize this is in part due to IHS access. IHS is underfunded, understaffed, has an aging infrastructure, and receives less per capita funding than other US federal health care services [33]. We suspect a weak health care system is not the sole contributor to the slightly better health we observed for SR-AIANs.

There are limitations to this study. First, we were unable to use the most current BRFSS data because detailed MR data is not available in the public use datasets after 2012. Second, though we used an MSA variable in the analyses to control for urban and rural differences, the MSA variable is only reported for individuals responding to BRFSS via landlines; consequently, there is no way to determine urban/rural residence for cell phone users. Inclusion of the MSA variable without indicating a missing category would have limited our sample to landline users potentially introducing bias since more SR-AIANs had missing MSA data. Additionally, we acknowledge that cell phone users and nonusers residing in urban and rural locations are likely heterogenous which can lead to bias. SR-AIANs had slightly more missing data on the income variable than MR-AIANs and SR-Whites. Excluding those with missing income would have excluded slightly more SR-AIANs than the other two groups, we therefore created a missing data category for non-responders. However, we do recognize that individuals who chose to report and chose not to report income are also heterogenous which may bias results. We recognize there may be many unmeasured factors that potentially contribute to the observed health differences between SR and MR-AIANs such as identity stress, discrimination related to health care access, culture, and environment contributions. Despite these limitations, our findings are consistent with and extend the literature related to the role of MR identification regarding risks, outcomes, and resulting health disparities.

## Conclusion

MR-AIANs are at greater risk for poor outcomes compared to SR-AIANs on several chronic conditions. Both are at greater risk than their SR-White counterparts for poor health across many identical health outcomes. Regardless of AIAN-SR or MR identification, these findings support the continued need to address health disparities among AIANs. We must also carefully consider national statistics that report SR-AIANs only in results. These analyses mirror the results of our earlier work on the burden of mental health problems associated with MR and SR identity. The underlying dynamics are not simple and must consider a wide range of lifestyle and environmental factors, some of which are undoubtedly linked to the social and psychological aspects of racial identity. Research on stress related to MR identity, discrimination in the health care systems, along with differences is access to care is needed to understand the complex health disparities that exist between SR and MR-AIANs.
